# Clinical features associated with the efficacy of chemotherapy in patients with glioblastoma (GBM): a surveillance, epidemiology, and end results (SEER) analysis

**DOI:** 10.1186/s12885-021-07800-0

**Published:** 2021-01-19

**Authors:** Jieqiong Wen, Wanbin Chen, Yayun Zhu, Pengbo Zhang

**Affiliations:** 1 Department of Anesthesiology, The Second Affiliated Hospital of Xi’an Jiaotong University, 157# West 5 Road, Xi’an, 710004 Shaanxi China; 2 Department of Marketing, The Johns Hopkins University Carey Business School, Baltimore, MD USA; 3 Department of Liver Surgery and Transplantation, Liver Cancer Institute, Zhongshan Hospital, Fudan University, Shanghai, China

**Keywords:** Glioblastoma, Chemotherapy, SEER, Survival

## Abstract

**Background:**

Glioblastoma (GBM) is a highly malignant brain tumor with poor survival and prognosis. Randomized trials have demonstrated that chemotherapy improves survival in patients with GBM. This study aims to examine the clinical characteristics that are potentially associated with the efficacy of chemotherapy and the risk factors of GBM.

**Methods:**

A total of 25,698 patients diagnosed with GBM were identified between 2004 and 2015 from the Surveillance, Epidemiology, and End Results (SEER). The clinical and demographic variables between groups were examined by Student’s t-test and Pearson’s chi-square test. GBM-specific survival (GBMSS) and overall survival (OS) were evaluated using the Kaplan-Meier method with the log-rank test. Univariable and multivariable analyses were also performed using the Cox proportional hazards model to identify statistically significant prognostic factors.

**Results:**

Patients who received chemotherapy had better overall survival (median OS 13 vs. Three months, HR = 1.9224, 95%CI 1.8571–1.9900, *p* < 0.0001) and better GBMSS (median GBMSS of 12 vs. Three months, HR = 1.9379, 95%CI 1.8632–2.0156, *p* < 0.0001), compared to patients who did not. Further subgroup analysis revealed that among patients who underwent chemotherapy, those who were younger, with a supratentorial tumor, received surgery, or radiotherapy had both improved OS and GBMSS. Age, race, tumor location, tumor size, and treatments were identified as independent prognostic factors by multivariable analyses for patients with glioblastoma.

**Conclusion:**

Patients with GBM who were younger (< 65 years), underwent surgery, or radiotherapy can benefit more from chemotherapeutic regimens. Age, race, tumor size, tumor location, surgery, radiotherapy, and chemotherapy were factors associated with the prognosis of patients with GBM.

**Supplementary Information:**

The online version contains supplementary material available at 10.1186/s12885-021-07800-0.

## Background

Glioblastoma (GBM), a WHO grade IV glioma and the most common primary malignant brain tumor in adults, accounts for 45.2% of malignant central nervous system (CNS) tumors with an incident rate of 4.32 per 100,000 in the United States [1, 2]. Due to the aggression and the high recurrence rate of GBM, the prognosis of this lethal disease is bleak, with a median survival of fewer than 2 years. Long-term survivors of glioblastoma encounter neurologic deficits and mental disorders, suffering from the reduced quality of life and substantial financial burden [3].

The current standard of care for newly diagnosed patients includes surgical resection, radiotherapy plus concomitant daily Temozolomide (TMZ), followed by adjuvant TMZ for 6 cycles, based on the results of a phase III clinical trial published in 2005 [4, 5]. The clinical trial showed that the median survival of patients with radiotherapy plus TMZ (14.6 months) was longer than that of patients with radiotherapy alone (12.1 months) and that the two-year survival rate was increased to 26.5% [6]. Besides, the benefits of combined therapy lasted throughout 5 years of follow-up [7]. Bevacizumab (BEV), another U.S. Food and Drug Administration (FDA) approved drug, is widely used for treatment in recurrent GBM. The survival benefits of TMZ and BEV have been widely explored in the clinical trials [8–12] and retrospective studies [13–19]. And, second-line chemotherapy options include nitrosoureas, lomustine, cyclophosphamide, irinotecan, and platinum-based regimens, etc. [20]. Despite the advancements in the therapeutic regimen, the improvement in the overall patient survival is still unsatisfactory. A phase III study showed that the addition of BEV to radiotherapy-TMZ did not result in survival benefits in patients with glioblastoma [21]. Blood-brain barrier (BBB), low selectivity, and side effects of drugs severely limit the efficacy of treatment [22].

The current care standard regimen is based on the outcomes from patients aged 18 to 70 years [6], while most patients diagnosed with GBM are older than 60 years with a median age of 64.0 years [23]. In the current study, we utilized the Surveillance, Epidemiology, and End Results (SEER) database to estimate the survival of patients with GBM of all ages receiving chemotherapy or not from 2004 to 2015, aim to explore the clinicopathological features affecting the efficacy of chemotherapy and the risk factors of GBM.

## Methods

### Data source

Data were obtained from SEER 18 registries Data, which was maintained by the National Cancer Institute (https://seer.cancer.gov/). The SEER program is a comprehensive source of population-based information and the largest available cancer dataset in the world, covering approximately 34.6% population of the U.S.

### Study population and variables

**Fig. 1:**
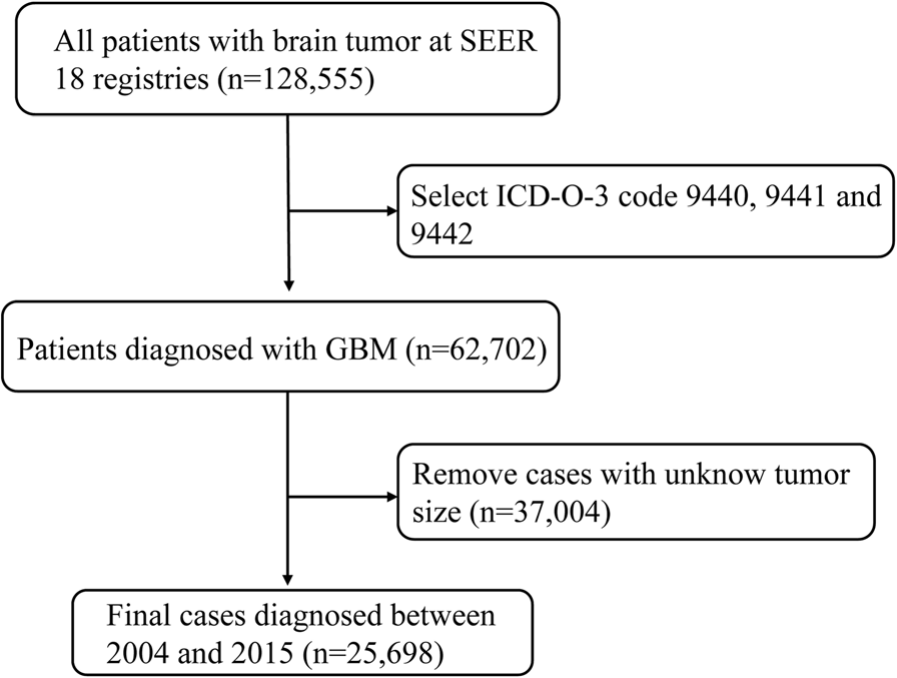
Flowchart for patient information retrieval from the Surveillance, Epidemiology, and End Results database

Data of patients diagnosed with GBM as primary cancer were extracted from the SEER database. Based on International Classification of Disease for Oncology, Third Edition (ICD-O-3), GBM cases were identified by histology codes 9440 (glioblastoma, NOS), 9441 (giant cell glioblastoma), and 9442 (gliosarcoma). Of 62,702 patients with a diagnosis of GBM between the year 2004 and 2015 were included in the SEER Registry, while those with unknown tumor size were excluded; 25,698 patients were eligible for inclusion into the present study (Fig. 1, Flowchart). Eligible patients were grouped according to chemotherapy status defined by chemotherapy recode. Age at diagnosis data was categorized as: young, < 18 years of age; adult, 18–65 years of age; old, ≥66 years of age. Tumor size was defined as the largest dimension or the diameter of the primary tumor. Tumor location was categorized as: supratentorial tumor (cerebrum, frontal lobe, temporal lobe, parietal lobe, occipital lobe), infratentorial tumor (ventricle, cerebellum, brain stem), and overlapping lesion of the brain. Overall survival (OS) was defined as the survival months from diagnosis to death regardless of the cause of death. GBM specific survival (GBMSS) was defined as the survival months from diagnosis to death due to GBM. The relationship between chemotherapy status and clinical characteristics, including age at diagnosis, gender, race, primary site, tumor size, and treatments was analyzed.

### Statistical analysis

The baseline characteristics of patients and treatments were described using summary statistics, with continuous variables being shown as mean ± standard deviation. Continuous variables were compared using the t-test, qualitative variables were compared using the chi-square test. Survival was estimated using the Kaplan–Meier method and differences between groups were estimated using the log-rank test. Hazard ratios (HRs) and 95% confidence intervals (CIs) were calculated by univariable and multivariable Cox proportional hazards models to assess the relative contribution of chemotherapy and other factors to survival after diagnosis of GBM. This study performed the statistical analyses in R software. All the tests above were 2-tailed, and a *p*-value of less than 0.05 was considered statistically significant.

## Results

### Demographics

Of 25,698 patients diagnosed with GBM between 2004 and 2015 in SEER were enrolled in this study, 18,066 patients received chemotherapy, and 7632 patients did not. The demographic characteristics of patients with chemotherapy or not are displayed in Table 1.

**Table 1:** Patients characteristics

Characteristic	Total, n	No chemotherapy, n	Received chemotherapy, n	*p*
	25,698	7632	18,066	
**Age at diagnosis, n (%)**				
Young	271 (1.05)	55 (0.72)	216 (1.20)	< 0.0001
Adult	14,134 (55.00)	2817 (36.91)	11,317 (62.64)	
Old	11,293 (43.95)	4760 (62.37)	6533 (36.16)	
**Gender, n (%)**				
Male	14,920 (58.06)	4119 (53.97)	10,801 (59.78)	< 0.0001
Female	10,778 (41.94)	3513 (46.03)	7265 (40.21)	
**Race, n (%)**				
White	22,963 (89.36)	6771 (88.72)	16,192 (89.63)	0.026
Black	1438 (5.60)	462 (6.05)	976 (5.40)	
American indian/alaska native	109 (0.42)	24 (0.31)	85 (0.47)	
Asian of pacific islander	1146 (4.46)	354 (4.64)	792 (4.38)	
Unknown	42 (0.16)	21 (0.28)	21 (0.12)	
**Primary site, n (%)**				
Supratentorial	20,170 (78.48)	5792 (75.89)	14,378 (79.59)	< 0.0001
Infratentorial	417 (1.62)	133 (1.74)	284 (1.57)	
Brain overlap	3803 (14.80)	1229 (16.11)	2574 (14.25)	
Unknown	1308 (5.10)	478 (6.26)	830 (4.59)	
**Size, n (%)**				
≤ 4 cm	9606 (37.38)	2661 (34.87)	6945 (38.44)	< 0.0001
> 4 cm	16,092 (62.62)	4971 (65.13)	11,121 (61.56)	
**Surgery, n (%)**				
No	5219 (20.31)	2598 (34.04)	2621 (14.51)	< 0.0001
Yes	20,436 (79.52)	5005 (65.58)	15,431 (85.41)	
Unknown	43 (0.17)	29 (0.38)	14 (0.08)	
**Radiation, n (%)**				
No	8883 (34.57)	5692 (74.58)	3191 (17.66)	< 0.0001
Yes	16,815 (65.43)	1940 (25.42)	14,875 (82.34)	

The median age at diagnosis for all patients was 63 years, 61 years for the chemotherapy cohort, and 71 years for the no chemotherapy cohort. Adult patients (18–65 years old) were the largest population in both the entire cohort (55%) and the chemotherapy cohort (62.64%), old patients (> 65 years old) constituted the highest proportion in no chemotherapy cohort (62.37%). Among all the patients, 58.06% were males, and 89.36% were white. The supratentorial region was the most common primary site of GBM (83%), followed by the overlapping brain area (14.80%) and infratentorial area (1.625%). Most of the patients had tumors larger than 4 cm (62.62%). Most patients received surgery (79.52%) and radiotherapy (65.43%). Compared to no chemotherapy cohort, the chemotherapy cohort was more likely to receive surgery (85.41% vs. 65.58%). Most patients in the chemotherapy cohort underwent radiotherapy (82.34%), while radiotherapy was less observed in no chemotherapy cohort (25.42%).

### Kaplan–Meier survival analysis

**Fig. 2:**
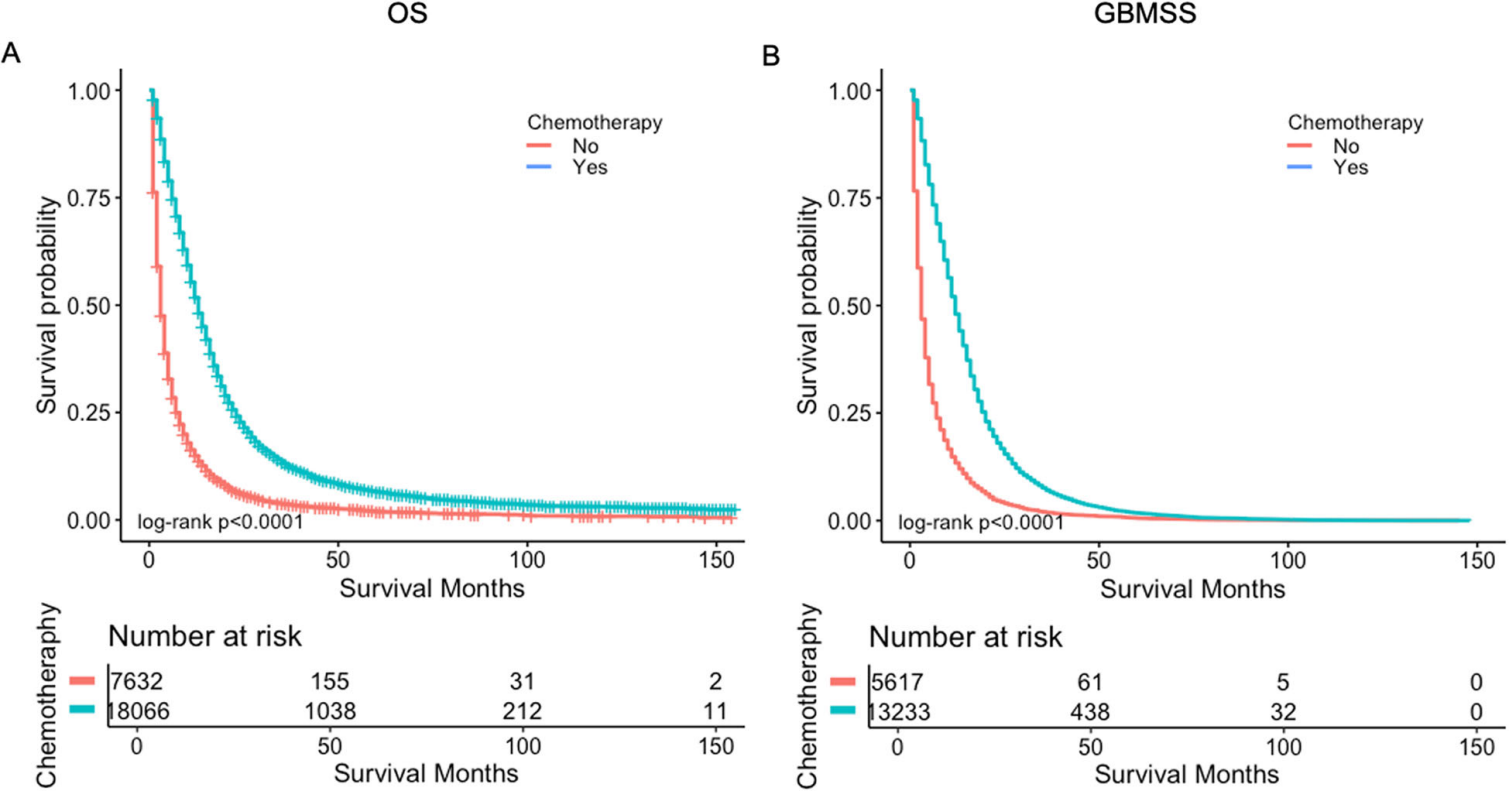
Survival curves of OS and GBMSS based on chemotherapy status

The median overall survival OS for the entire cohort was 9 months, the chemotherapy cohort had better OS than no chemotherapy cohort, with a median of 13 months versus 3 months (*p* < 0.0001) (Fig. 2a), and better GBMSS, with a median of 12 months versus 3 months (*p* < 0.0001) (Fig. 2b).

**Fig. 3:**
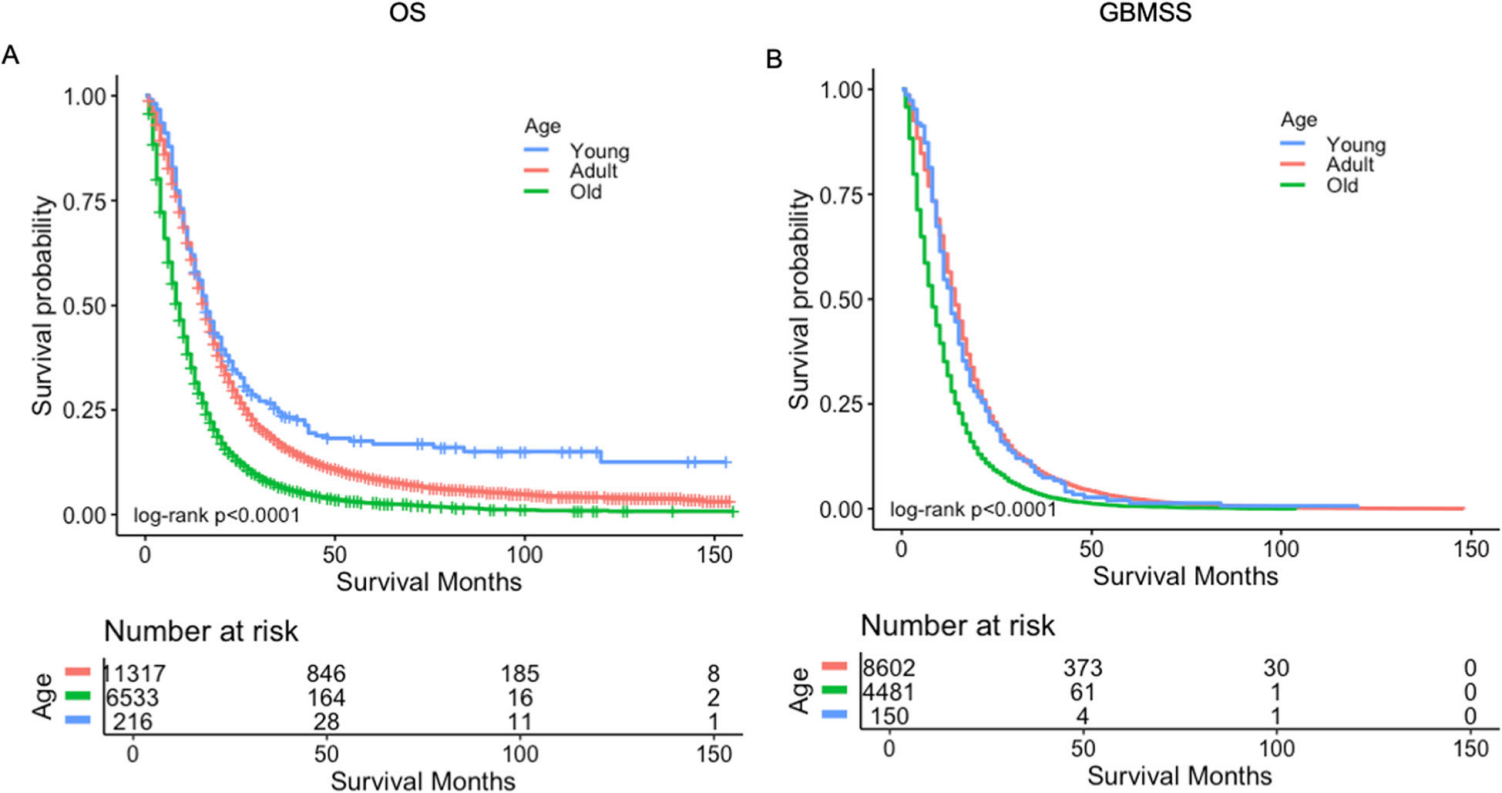
Survival curves of OS and GBMSS for patients with chemotherapy based on age

**Fig. 4:**
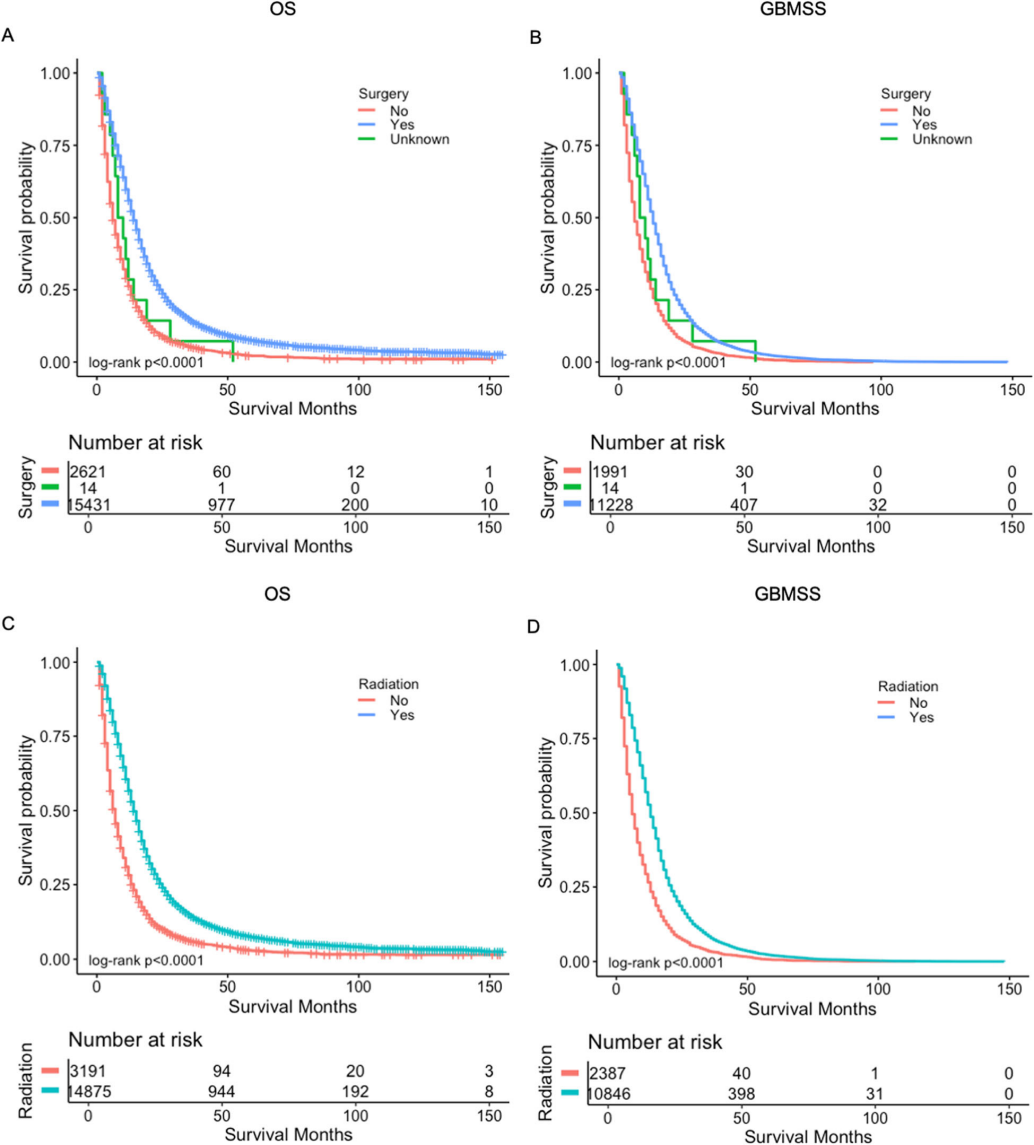
Survival curves of OS and GBMSS for patients with chemotherapy based on surgery and radiotherapy

In order to figure out the factors associated with the efficacy of chemotherapy, we performed subgroup analysis among the 18,066 patients who received chemotherapy. We observed that those who were at age < 18 years benefited most from chemotherapy, followed by patients aged 18–65 years, and patients> 65 years (median OS: 16, 15, and 9 months, respectively, *p* < 0.0001; median GBMSS: 14, 13, and 8 months, respectively, *p* < 0.0001) (Fig. 3a, b). Patients who received surgical resection were also observed to have better survival than those who did not (median OS: 14 vs. 6 months, *p* < 0.0001; median GBMSS: 13 vs. 6 months, *p* < 0.0001) (Fig. 4a, b). Further, radiation therapy was also contributing to the improved survival for patients who received chemotherapy, compared with those who did not (median OS: 14 vs. 7 months, *p* < 0.00001; median GBMSS: 13 vs. 6 months, *p* < 0.0001) (Fig. 4c, d). We also explored the effects of other treatments for patients who did not receive chemotherapy and found that patients had better OS and GBMSS after surgery or radiation therapy (Additional file 1: Fig. S1). When compared the different combinations of treatments, for patients who received surgery, patients who also received chemotherapy and radiotherapy had the best GBMSS and OS. For patients who did not receive surgery, they still had survival benefit from the combination treatment of chemotherapy and radiotherapy or chemotherapy alone. While, for patients who only received radiotherapy, the number of cases was too small (Additional file 1: Fig. S2).

### Cox regression analysis of survival

To further figure out the clinical variables associated with GBM, the Cox proportional hazards model was applied to the analysis. Univariable analysis of GBMSS and OS is shown in Table 2. Compared to young patients, patients aged between 18 and 65 years had worse OS (HR 1.278, 95% CI 1.119–1.460, *p* = 0.0003), while the difference in GBMSS was not significant (*p* = 0.8270). Patients aged over 65 years old had the worst GBMSS (HR 1.98, 95% CI 1.7172–2.284, *p* < 0.0001) and OS (HR 2.632, 95% CI 2.304–3.007, *p* < 0.0001). White, infratentorial location, and tumor size larger than 4 cm were all associated with poorer GBMSS and OS (HR > 1, *p* < 0.01). Patients who did not receive surgery had decreased GBMSS and OS (HR = 2.186, 95%CI, 2.109–2.265, *p* < 0.0001; HR = 2.287, 95%CI, 2.216–2.360, *p* < 0.0001, respectively) compared to those who received surgery. Patients who did not undergo radiotherapy were at increased risk of worse GBMSS and OS (*p* < 0.0001). Patients did not receive chemotherapy had worse GBMSS (HR 2.617, 95% CI 2.545–2.691, *p* < 0.0001) and OS (HR 2.571, 95% CI 2.438–2.599, *p* < 0.0001) (Table 2). The multivariable Cox analysis also evidenced these results and showed that age, gender, race, tumor size, tumor location, and treatments were significantly associated with the survival of GBM patients (Table 3).

**Table 2:** Univariate Analysis of Prognostic Factors of GBMSS and OS in GBM

Characteristic	HR	GBMSS	*p*	HR	OS	*p*
		95%CI			95%CI	
**Age at diagnosis**						
Young	1			1		
Adult	1.016	0.8814–1.171	0.8270	1.278	1.119–1.460	0.0003
Old	1.98	1.7172–2.284	< 0.0001	2.632	2.304–3.007	< 0.0001
**Gender**						
Female	1			1		
Male	0.950	0.923–0.978	0.0005	1.003	0.9769–1.029	0.8470
**Race**						
White	1			1		
Black	0.9173	0.861–0.9773	0.0076	0.9072	0.8578–0.9594	0.0007
American indian/alaska native	0.9074	0.7295–1.1288	0.3831	0.9158	0.7486–1.1203	0.3922
Asian of pacific islander	0.8523	0.7945–0.9143	< 0.0001	0.8251	0.7744–0.8791	< 0.0001
Unknown	1.2114	0.7974–1.840	0.3687	0.6008	0.4174–0.8647	0.0061
**Primary site**						
Supratentorial	1			1		
Infratentorial	1.185	1.1385–1.233	< 0.0001	1.206	0.8289–1.1639	< 0.0001
Brain overlap	1.099	0.9628–1.255	0.1620	1.043	0.9586–0.9306	0.4680
**Size**						
≤ 4 cm	1			1		
> 4 cm	1.08	1.047–1.114	< 0.0001	1.079	1.05–1.109	< 0.0001
**Surgery**						
Yes	1			1		
No	2.186	2.109–2.265	< 0.0001	2.287	2.216–2.360	< 0.0001
Unknown	1.814	1.289–2.552	0.0006	1.761	1.296–2.392	0.0003
**Radiation**						
Yes	1			1		
No	2.41	2.338–2.485	< 0.0001	2.514	2.447–2.583	< 0.0001
**Chemotherapy**						
Yes	1			1		
No	2.617	2.545–2.691	< 0.0001	2.571	2.438–2.599	< 0.0001

*Abbreviations*: *HR* Hazardous Ratio, *CI* Credential Interval, *OS* overall survival, *GBMSS* GBM specific survival

**Table 3:** Multivariate Analysis of Prognostic Factors of GBMSS and OS in GBM

Characteristic	HR	GBMSS	*p*	HR	OS	*p*
		95%CI			95%CI	
**Age at diagnosis**						
Young	1			1		
Adult	1.1050	0.9530–1.2812	0.1860	1.3528	1.1792–1.5520	< 0.0001
Old	1.9541	1.6844–2.2669	< 0.0001	2.4902	2.1694–2.8584	< 0.0001
**Gender**						
Female	1			1		
Male	1.0135	0.9836–1.0443	0.3807	1.0545	1.0267–1.0830	< 0.0001
**Race**						
White	1			1		
Black	0.9457	0.8856–1.010	0.0962	0.9023	0.8515–0.9563	0.0052
American indian/alaska native	0.8635	0.6879–1.084	0.2059	0.9563	0.7745–1.1808	0.6781
Asian of pacific islander	0.7924	0.7368–0.8521	< 0.0001	0.7918	0.7417–0.8453	< 0.0001
Unknown	0.9990	0.6441–1.5495	0.9965	0.4843	0.3319–0.7066	0.0017
**Primary site**						
Supratentorial	1			1		
Infratentorial	1.0966	1.0532–1.1419	< 0.0001	1.1105	1.0710–1.1514	< 0.0001
Brain overlap	1.0451	0.9267–1.1787	0.4721	1.0159	0.9155–1.1274	0.7663
**Size**						
≤ 4 cm	1			1		
> 4 cm	1.1379	1.1033–1.1737	< 0.0001	1.1409	1.1099–1.1727	< 0.0001
**Surgery**						
Yes	1			1		
No	1.4743	1.3967–1.5563	0.0014	1.4000	1.3364–1.4666	< 0.0001
Unknown	1.2474	0.8749–1.7784	0.2220	0.9295	0.6778–1.2746	0.6500
**Radiation**						
Yes	1			1		
No	1.4125	1.3425–1.4862	< 0.0001	1.5072	1.4420–1.5754	< 0.0001
**Chemotherapy**						
Yes	1			1		
No	1.9379	1.8632–2.0156	< 0.0001	1.9224	1.8571–1.9900	< 0.0001

*Abbreviations***:** *HR* Hazardous Ratio, *CI* Credential Interval, *OS* overall survival, *GBMSS* GBM specific survival

## Discussion

In the current SEER-based study, we analyzed the clinicopathological features that are potentially associated with the efficacy of chemotherapy to identify certain patient populations that might be more likely to benefit from chemotherapy. Also, we explored the prognostic factors of GBM by univariate and multivariate Cox analyses. We observed that chemotherapy was associated with improved OS and GBMSS. Besides, the benefits of chemotherapy were greater in patients who younger than 65 years old or underwent additional treatments. Age at diagnosis, race, tumor size, tumor location, surgery, radiotherapy, and chemotherapy were the major factors of prognosis.

The validated chemotherapeutic agents TMZ and BEV were approved by the FDA in 2005 and 2009 respectively. The population enrolled in our study was diagnosed with GBM between 2004 and 2015. The median survival of patients who received chemotherapy reported in this study was significantly longer than patients who did not. The benefit of chemotherapy has been proved in previous studies. However, it has generally accepted the improvements provided by the current treatment are limited. The progression-free survival of patients with lomustine plus BEV was 2.7 months longer than lomustine alone, while the median OS of patients with the addition of BEV was not increased significantly compared to lomustine alone [24]. Dose-dense TMZ for newly diagnosed GBM did not improve overall survival or progression-free survival compared to standard TMZ [25]. One of the reasons is that some cases are resistant to TMZ, and O6 -methylguanine-DNA methyltransferase (MGMT) mainly contributes to the TMZ chemoresistance [26]. Toxicity of drugs and BBB are the potential factors that limit the efficacy of chemotherapy. A cumulative dose of TMZ increases the risk of lymphopenia [27]. BEV may cause toxicity, bleeding events, stroke, and wound-healing complications [28]. Although BBB is disrupted partly, increased expression of pro-angiogenic factors and increased interstitial fluid pressure still limit small-molecule drugs through the BBB and towards the site of the tumor [29]. Local drug delivery vehicles, including carmustine (BCNU)-loaded polyanhydride wafers, convection-enhanced delivery, microsphere formulations, or drug-loaded nanoparticles, and intraventricular delivery, intrathecal delivery, intranasal delivery are used for accelerating the delivery of therapeutic compounds to the brain [30]. In addition to promising new drug delivery materials, the optimal therapeutic regimen is being determined. A study analyzed data from the French Brain Tumor DataBase, compared to standard 6 cycles, patients received adjuvant TMZ beyond 6 cycles had improved survival [31]. The delay in the initiation of chemotherapy and radiation following resection longer than 6 weeks was associated with worse OS [32].

The current care standard regimen is based on the study involved patients under 70 years, while the median age of GBM patients is 64.0 years and the incident rate of GBM appears to correlate with increased age [23]. There was increased use of chemotherapy and radiotherapy from 40.3% in 2004 to 59.8% in 2012 among patients aged 70 years and older [33]. Therefore, the efficacy of adjuvant therapy in the elderly population needs to be discussed. In our study, all ages were included and divided into three age groups with the median age 63 years. Elderly patients aged 65 years or over had the worst median OS (5 months) and GMBSS (5 months) among all age groups. Although young patients benefited most from chemotherapy, the survival of elderly patients was also improved by chemotherapy. A trial involving 562 patients 65 years or older showed that radiotherapy plus TMZ had longer median OS compared to patients with radiotherapy alone [8]. Thus, chemotherapy should be considered as an effective treatment for all ages.

We found that there was a relationship between tumor location and survival, and most GMB was supratentorial tumor with better survival than other locations. A study showed that 61% primary gliomas occur in the four lobes of the brain, frontal (25%), temporal (20%), parietal (13%), and occipital region (3%), brainstem and cerebellum only accounts for 1.2 and 0.9%, respectively [34]. Patient characteristics, histologic features, and genomic profiles are different between supratentorial glioblastoma and cerebellar glioblastoma [35]. The most impacted function region was eloquent cortex [36]. Patients with tumors in the right deep periventricular white matter region had poor survival [37]. Tumor location impacts the extent of resection and postoperative tumor volume. A study involved patients younger than 20 years old suggests that aged 0–4 years, infratentorial tumor location, and subtotal resection were associated with higher mortality [38]. In addition to tumor location, our study found that the diameter of the tumor over 4 cm was associated with poorer survival. A clinical trial showed that the median preoperative tumor volume was 24.9 cm^3^, and patients aged over 60 years showed significantly increased tumor volume which was 30.9 cm^3^. Postoperative tumor volume remained was a significant prognostic factor. The median postoperative tumor volume was 0.1 cm^3^; the difference in residual tumor volumes between older and younger patients was not significant [39].

In our study population, most patients received multiple treatments. Among patients who received chemotherapy, 85.41% underwent surgery and 82.34% underwent radiotherapy. Moreover, surgery and radiotherapy were prognostic factors for patients with GBM. We observed that surgery or radiotherapy improved survival when combined with chemotherapy, compared to chemotherapy alone. The high infiltration nature of GBM cells makes it difficult to achieve complete eradication of the primary tumor and leads to a high recurrence rate. Surgery is the pivotal treatment for GBM, the extent of resection (EOR) and residual volume are significantly associated with prognosis. Increased EOR and reduced residual volume were associated with longer survival and delayed recurrence of patients who received adjuvant therapies, including TMZ and radiation therapy [40]. Radiotherapy is involved in the standard regimen and is an effective treatment for unresectable GBM. Some studies have shown that the chemoradiation strategy is more effective than radiotherapy alone [6, 9, 41].

In our study, gender was one of the prognostic factors for OS. Females with GBM often had a better outcome than males, gender disparity involved mechanisms, and immune function contribute to different outcomes [42, 43]. Gender differences also reflect in different molecular subtypes of GBM, mesenchymal subtype, proneural subtype, and neural subtype most occur in males, while the classical subtype is equally prevalent in males and females. We found that white patients were associated with high risk of death. A study showed that non-Hispanic whites had higher incidence and lower survival rates [44].

There are potential limitations to our study. First, information about chemotherapy is deficient. According to the SEER data, there exists a certain number of patients whose chemotherapy status is not sure, and that may cause misclassification. Although some results are consistent with previous studies, there still have some biases inevitably. Second, the information about chemotherapy drugs and specific treatment is less detailed, whether targeted agents were involved in the therapy is unknown. Since targeted therapies could reduce unwanted toxicity and are more effective, patients who received targeted therapies or not may have different survival benefits. Finally, regarding treatments, we did not further discuss the detailed treatment modality, the radiotherapy sequence with surgery and chemotherapy information of individual patients.

In summary, our study supports the idea that combined therapy, namely, surgery or radiotherapy plus chemotherapy, might bring about more survival benefits than chemotherapy, surgical resection, or radiotherapy alone, which, in clinical settings, suggests that after considerate assessments, clinicians should encourage eligible patients to receive chemotherapy with surgery or radiotherapy to maximize their survival benefits.

## Conclusion

Patients with GBM who were younger (< 65 years), underwent surgery, or radiotherapy can benefit more from chemotherapeutic regimens. Age, race, tumor size, tumor location, surgery, radiotherapy, and chemotherapy were factors associated with the prognosis of patients with GBM.

## Supplementary Information

**Additional file 1: Fig. S1**. Survival curves of OS and GBMSS for patients did not receive chemotherapy based on surgery and radiotherapy.

**Additional file 2: Fig. S2**. Survival curves of OS and GBMSS for patients based on different treatment combinations.

## Data Availability

The data were abstracted from an open database, the Surveillance, Epidemiology, and End Results (SEER) 18 Registries Data (https://seer.cancer.gov/).

## Abbreviations

GBM: Glioblastoma
SEER: Surveillance, Epidemiology, and End Results
GBMSS: GBM-specific survival
OS: Overall survival
CNS: Central nervous system
TMZ: Temozolomide
BEV: Bevacizumab
FDA: U.S. Food and Drug Administration
HRs: Hazard ratios
CIs: Confidence intervals
BBB: Blood-brain barrier
MGMT: O6 -methylguanine-DNA methyltransferase
EOR: Extent of resection
