# Supplementary figures and images for: Clinical features associated with the efficacy of chemotherapy in patients with glioblastoma (GBM): a surveillance, epidemiology, and end results (SEER) analysis
Source: BMC Cancer. 2021 Jan 19;21:81. doi: 10.1186/s12885-021-07800-0 (PMC7816395; doi:10.1186/s12885-021-07800-0)

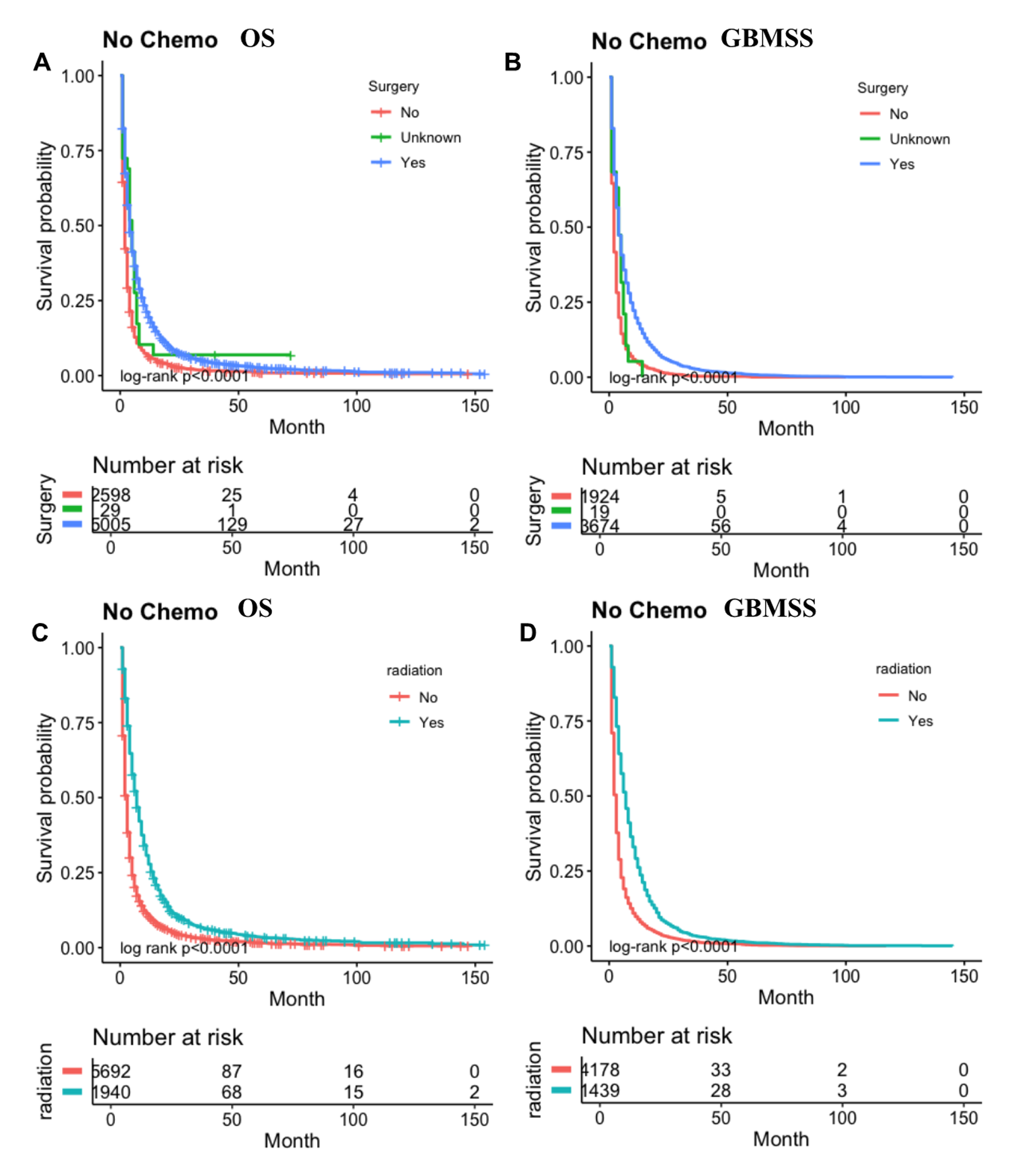

Supplement: Supplementary file 1 — Additional file 1: Fig. S1. Survival curves of OS and GBMSS for patients did not receive chemotherapy based on surgery and radiotherapy. [file 12885_2021_7800_MOESM1_ESM.tif]

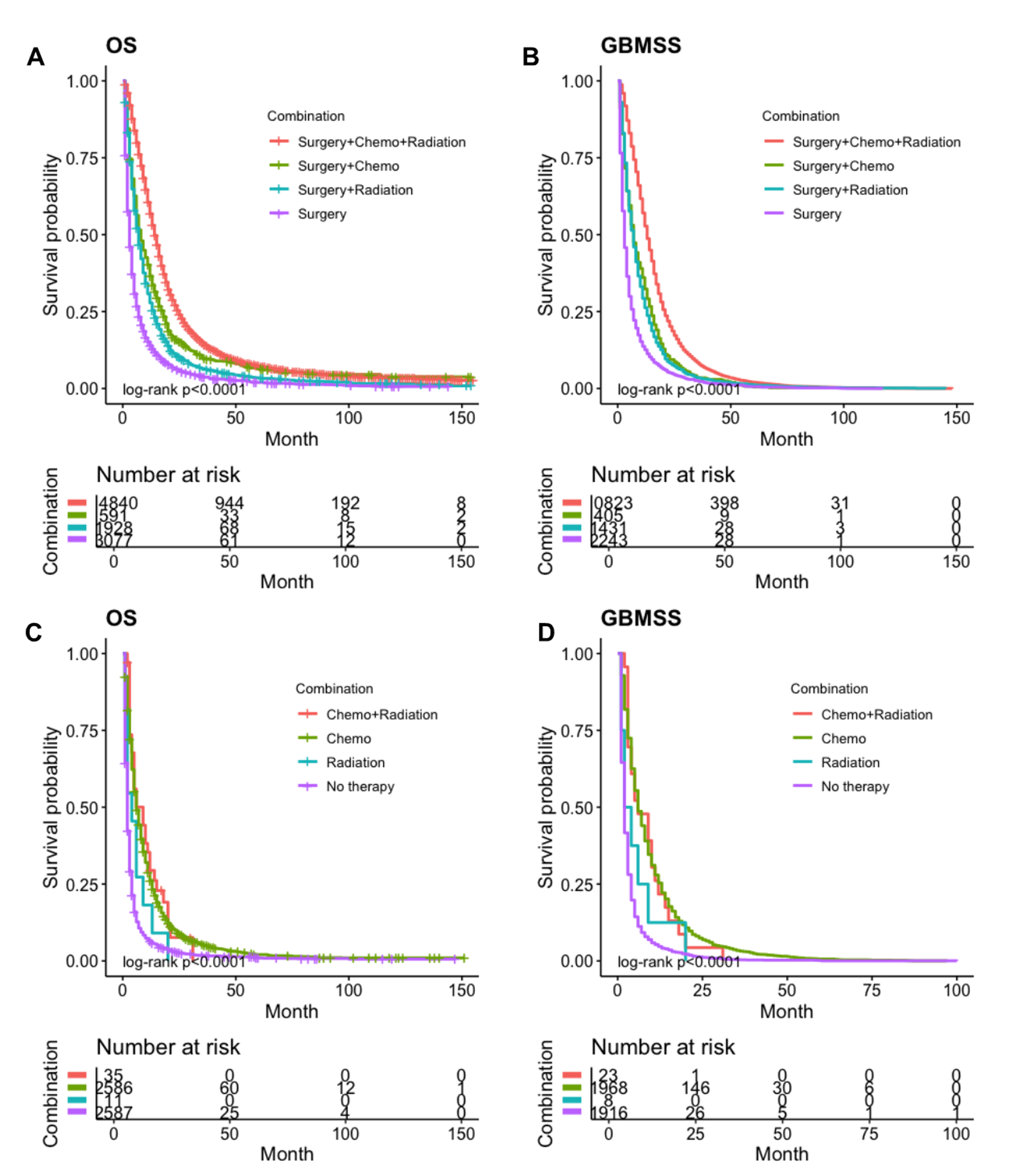

Supplement: Supplementary file 2 — Additional file 2: Fig. S2. Survival curves of OS and GBMSS for patients based on different treatment combinations. [file 12885_2021_7800_MOESM2_ESM.tif]
